# How place shapes genital herpes simplex distribution in South Korea: a Bayesian spatial analysis using National Health Insurance Service data

**DOI:** 10.1186/s12889-025-24171-4

**Published:** 2025-09-02

**Authors:** Joonsu Jang, Seyul Park, Byung Chul Chun

**Affiliations:** 1 Department of Preventive Medicine, Korea University College of Medicine, Goryeodae-Ro 73, Seongbuk-Gu, Seoul, 02841 Republic of Korea; 2 Department of Public Health, College of Medicine, Korea University Graduate School, Goryeodae-Ro 73, Seongbuk-Gu, Seoul, 02841 Republic of Korea

**Keywords:** Spatial epidemiology, Sexually transmitted infections, Bayesian spatial modeling, South Korea, Genital herpes simplex

## Abstract

**Background:**

Genital herpes simplex (GHS) infections have increased in South Korea over the past decade, yet the geographic distribution of GHS incidence rates remains poorly understood. This study examined the geographic distribution of GHS incidence rates across South Korea and identified place-specific risk factors to inform targeted prevention strategies.

**Methods:**

Using the Korean National Health Insurance Service data from 2019, we analyzed age-standardized GHS incidence rates across 250 municipalities in South Korea. Spatial autocorrelation was assessed using Global Moran's I and hot spots were identified using the Getis–Ord Gi* analysis. Bayesian hierarchical regression models were used to quantify the associations between regional risk factors and GHS risk while accounting for spatial dependence. The area-specific posterior mean was used to map the relative risk (RR) of GHS.

**Results:**

In total, 182,289 patients with GHS were identified. Significant positive spatial autocorrelation was observed (Moran's I = 0.431, *p* < 0.001), with distinct hot spots concentrated in the Seoul Capital Area. After adjusting for covariates and spatial effects, the proportion of single-person households (RR = 1.026, 95% credible interval (CrI) = 1.017–1.035) and sexual violence risk (2nd quartile: RR = 1.106, 95% CrI = 1.018–1.201; 3rd quartile: RR = 1.095, 95% CrI = 1.003–1.195) were significant place-based risk factors associated with higher GHS incidence rates. High-risk areas for GHS were found in the Seoul Capital Area and some mid-southern cities.

**Conclusions:**

This study reveals distinct geographic patterns in GHS incidence rates that are not fully explained by demographic composition alone. The significant associations with household structure and sexual violence risk suggest that place-specific social contexts influence GHS incidence rates. These findings highlight the importance of spatially targeted interventions to address contextual vulnerabilities in regions with high GHS incidence rates.

**Supplementary Information:**

The online version contains supplementary material available at 10.1186/s12889-025-24171-4.

## Background

Sexually transmitted infections (STIs) pose a significant global public health challenge, with an estimated 374 million new infections worldwide each year [1]. Among these infections, genital herpes simplex (GHS) infections are one of the most prevalent chronic STIs, affecting approximately 491 million people globally [2]. GHS is caused by herpes simplex virus (HSV) types 1 and 2 and results in painful genital sores, with HSV-2 being responsible for most genital infections [3]. GHS poses unique public health challenges due to its chronic nature, potential for asymptomatic transmission, and lifelong persistence, with latent periods punctuated by recurrent symptomatic episodes [4]. Unlike other STIs, GHS has no cure with available treatments only managing symptoms and possibly reducing transmission risk. The pathogenesis of GHS involves initial infection of epithelial cells, followed by lifelong latency in sensory neurons, allowing the virus to evade immune clearance and persist indefinitely in the host [3]. This ability to establish latency contributes to the persistent global burden of HSV infection despite ongoing awareness campaigns and prevention efforts.

According to the South Korean STI sentinel surveillance data, the reported cases of major STIs, including chlamydia, gonorrhea, syphilis, and GHS, have shown increasing trends since 2010. GHS infections demonstrated a particularly dramatic increase. In particular, reported GHS cases have increased more than sevenfold over the past decade, increasing from 1,572 cases in 2010 to 11,608 cases in 2019 [5]. A previous epidemiological study in South Korea documented a rapid increase in HSV-2 seroprevalence, particularly among adults in their 30 s in the southern regions of the country [6]. Despite this increasing burden, there remains a substantial knowledge gap regarding spatial epidemiology of GHS infections across the country. Most existing research on GHS in South Korea has focused primarily on clinical aspects, diagnostic methods [7, 8], or nationwide trends without examining specific geographic variations in transmission patterns [6]. Understanding the geographic distribution of GHS and identifying high-risk clusters are essential for developing targeted interventions and resource allocation strategies, as demonstrated in studies of other infectious diseases in South Korea [9, 10] and internationally [11]. Although spatial analytical methods have demonstrated their utility in identifying place-specific risk factors for various health outcomes, they have rarely been applied to STI epidemiology in South Korea.

Spatial epidemiology provides a critical framework for understanding geographic variations in disease distribution and identifying place-specific risk factors for transmission. This approach is particularly valuable for STIs such as GHS, where transmission dynamics are influenced by spatial proximity, social networks, and other local contextual factors that vary by location [12]. Previous spatial analyses of STIs in various countries have consistently revealed significant geographic clustering, which was often associated with urbanization and socioeconomic conditions. For example, studies in Portugal identified pronounced spatial heterogeneity in STI distribution, with clustering in specific regions, underscoring the value of geographic approaches for targeting prevention efforts [12]. In the United States, spatial analyses have revealed persistent geographic patterns in STIs associated with regional socioeconomic factors and demographic composition [13]. Similarly, a recent analysis in Ethiopia confirmed spatial clustering of STI cases across regions [14]. These spatial approaches have increasingly incorporated advanced statistical methods, including Bayesian hierarchical models, to account for spatial autocorrelation and generate more robust estimates of geographic risk factors while quantifying the uncertainty in spatial patterns.

This study aimed to address these knowledge gaps by conducting a nationwide spatial epidemiological analysis of GHS incidence rates across South Korea at the municipal level. Specifically, we sought to (1) characterize the demographic and geographic distributions of GHS incidence rates; (2) identify significant spatial clusters and patterns; and (3) quantify the associations between regional risk factors and GHS incidence rates while accounting for spatial dependence. By integrating nationwide health insurance data with place-specific factors and applying Bayesian spatial regression models, this study provides new insights into the spatial dynamics of GHS in South Korea.

## Methods

### Study design and setting

This study conducted an ecological spatial analysis to examine the geographic distribution and regional determinants of GHS incidence rates across South Korea in 2019. Figure S1 illustrates the study workflow. The study used the 2019 dataset, which was the most recent complete dataset available at the time this study was initiated. We analyzed data at the municipal level, which represents the smallest administrative division in the country for which comprehensive health data are publicly available. South Korea comprises 250 municipalities (si, gun, and gu) nested within 17 provinces and metropolitan cities, creating a hierarchical administrative structure. Geographic data for municipal boundaries were obtained from the National Spatial Geographic Information Service (https://sgis.kostat.go.kr).

### Data sources and variables

The number of GHS infection cases in 2019 was obtained from the Korean National Health Insurance Service (KNHIS) database [15]. These cases were identified using the International Statistical Classification of Diseases, 10th Revision, code A60 in the KNHIS records. The KNHIS, South Korea’s single insurer under mandatory health insurance for all Korean citizens, provides data through healthcare utilization claims that are considered representative of the national population [16]. The eligibility information within the KNHIS data is aggregated based on patient residence [17]. To adjust for differences in age structure across municipalities, indirect age-standardized incidence rates of GHS (GHSsir) per 100,000 population were computed, using the 2019 South Korean population as the standard. All subsequent regional clustering and Bayesian spatial analyses were conducted using these age-adjusted rates.

The selection of explanatory variables was based on previous STI studies that have demonstrated their relevance as determinants of STIs. The explanatory variables included multiple domains (Table 1): health behavior variables [18–20], welfare-related factors [21–23], health condition variables [24–27], socioeconomic variables [28–30], healthcare accessibility [31], adult entertainment establishments (count per municipality) [32], and the risk of sexual violence [33, 34]. The sexual violence risk variable was categorized into quartiles. Additionally, demographic covariates including the population density and sex ratio of each municipality were included to adjust for the underlying population structure. Except for the risk of sexual violence [33], all variables were obtained from national sources such as the Korean Community Health Survey (KCHS) [35], the Korean Statistical Information Service [36], and the public data portal (https://www.data.go.kr/en/inden.do). The variables collected from the KCHS (Table 1), originally at the individual level, were aggregated to the municipal level and converted into age‐standardized rates to correct for differences in age‐group composition across regions. All variables were aggregated at the municipal level. These explanatory variables were included in the final statistical model through a variable selection process, which is described in the Statistical analysis below.

**Table 1:** Description of study variables and data sources for genital herpes simplex analysis in South Korea, 2019

Variables	Description	Source (year)
Genital herpes simplex cases (N)	Patients with genital herpes simplex in 2019 by municipality	KNHIS^a^ (2019) [15]
Health behavior		
Exercise and physical activities (%)	Proportion of people in each municipality who engaged in moderate physical activity at least 30 min a day, 5 days a week, in the past week that made them feel slightly more tired or short of breath than usual	KCHS^b^ (2019) [35]
Current smoking (%)	Proportion of current smokers (“daily smokers” or “occasional smokers”) who have smoked five or more packs (100 cigarettes) in their lifetime (so far) per municipality	KCHS (2019)
Alcohol consumption (%)	Proportion of people who drank alcohol at least once a month in the past year per municipality	KCHS (2019)
High-risk drinking (%)	Proportion of people who drank seven or more drinks (or about five cans of beer) on a single occasion for men and five or more drinks (or about three cans of beer) for women at least twice a week in the past year per municipality	KCHS (2019)
Welfare-related variables		
Social welfare facilities density	Number of social welfare facilities per 100,000 population per municipality	KOSIS^c^ (2019) [36]
Share of municipal budget on social welfare (%)	Total budget for social welfare and healthcare in the current year per municipality	KOSIS (2019)
Subjective health perception (%)	Proportion of individuals who reported their health status is “very good” or “good” on a typical day per municipality	KCHS (2019)
EQ-5D index	European Quality of Life-5 Dimensions: an index that synthesizes a technical system of five dimensions of health-related quality of life (mobility, self-care, daily activities, pain/discomfort, and anxiety/depression) per municipality	KCHS (2019)
Healthy living practice (%)	Proportion of people who practice nonsmoking, moderate drinking, and walking per municipality	KCHS (2019)
Health Conditions		
Prevalence of depression (%)	Proportion of people with a total depression screening tool (PHQ-9) score of 10 or higher per municipality	KCHS (2019)
Prevalence of obesity (%)	Proportion of people with a body mass index (kg/m^2^) of 25 or higher per municipality	KCHS (2019)
Prevalence of diabetes (%)	Proportion of people aged ≥ 30 years who have been diagnosed with diabetes by a doctor per municipality	KCHS (2019)
Prevalence of hypertension (%)	Proportion of people aged ≥ 30 years who have been diagnosed with high blood pressure by a doctor per municipality	KCHS (2019)
Socioeconomic variables		
Low educational attainment (%)	Proportion of people aged ≥ 6 years with a high school diploma or less as their highest level of education per municipality	KOSIS (2015)
Single-person household (%)	Proportion of people in single-person households per municipality	KOSIS (2019)
Divorce (%)	Number of divorces in a year divided by the mid-year population in that year per 1,000 population per municipality	KOSIS (2019)
Financial autonomy (%)	Ratio of local taxes and nontax revenue to total general account revenue, indicating a municipality's self-financing capacity	KOSIS (2019)
Healthcare accessibility		
Doctors per 1,000 population	Number of doctors (medical doctors, Korean medicine doctors, and dentists) working in healthcare organizations per 1,000 population, per municipality	KOSIS (2019)
Unmet medical facilities (%)	Proportion of individuals who were unable to consult a doctor (excluding dentist) when needed in the past year per municipality	KCHS (2019)
Adult entertainment and sexual violence		
Adult entertainment establishments per 10,000 population	Number of establishments per 10,000 population that employ entertainment workers or have entertainment facilities, such as stand bars, room salons, and karaoke clubs, where food is prepared and sold primarily with alcoholic beverages	Public data portal (2019) (https://www.data.go.kr/)
Sexual violence risk	Sexual violence risk converted to quartiles based on the Crime Risk Assessment Tool applied to 250 municipalities	Park et al. (2014) [16]
Covariates		
Population density (N/1,000km^2^)	Population (N) in 2019 per 1,000 km^2^ per municipality	KOSIS (2019)
Sex ratio (%)	Number of men per 100 women per municipality	KOSIS (2019)

^a^KNHIS, Korean National Health Insurance Service

^b^KCHS, Korea Community Health Survey

^c^KOSIS, Korea Statistical Information Service

### Statistical analysis

The spatial autocorrelation of GHSsir was examined using Global Moran's I statistic with 999 Monte Carlo simulations to determine whether the geographic distribution of GHSsir was clustered or dispersed [37]. The Moran's I value ranges from −1 to + 1, with positive values indicating spatial clustering where similar values are near each other, negative values suggesting spatial dispersion where dissimilar values cluster together, and values near zero indicating spatial randomness. To identify specific geographic clusters of GHSsir, we conducted the Getis–Ord Gi* hot spot analysis using the GHSsir per 100,000 people [38]. We used the Getis–Ord Gi* approach instead of the local indicator of spatial autocorrelation because the Gi* statistic directly identifies clusters of high incidence rates (hot spots) and low incidence rates (cold spots), which are straightforward to interpret and visualize for our purposes [39]. The Getis–Ord Gi* calculates a z-score for each municipality by comparing the local averages to the global averages. High positive z-scores (> 1.96) indicate statistically significant hot spots (clusters of high values), whereas low negative z-scores (< −1.96) indicate cold spots (clusters of low values). Global Moran’s I and Getis–Ord Gi* statistics were computed using the R-package ‘spdep’ [40]. The resulting hotspot map was created with the R-package ‘tmap'[41], using significance levels of 90%, 95%, and 99% to indicate clustering intensity [42].

A Bayesian hierarchical model was used to quantify the associations between regional risk factors and the risk of GHS, while accounting for spatial dependence. This study chose a Bayesian modeling approach because it effectively accommodates spatially correlated count data, addressing the limitations of traditional regression models in handling spatial autocorrelation [43].

The observed number of GHS cases in each municipality, Y_i_, was analyzed. A dispersion test on a preliminary Poisson model in R (package ‘AER’) indicated that the dispersion parameter was significantly greater than unity (*p* < 0.001), confirming overdispersion [44]. Consequently, negative binomial regression models were fitted rather than Poisson models. Specifically, two Bayesian spatial models were implemented: the intrinsic conditional autoregressive (ICAR) model and the Besag–York–Mollié (BYM) model [45]. Both Bayesian hierarchical models incorporate spatial random effects to account for spatial dependence, potentially improving the accuracy of risk factor effect estimates.

Let Y_i_ be the number of observed GHS cases in municipality i (i = 1, 2, …, 250). Y_i_ is assumed to follow a negative binomial distribution to account for potential overdispersion:

1 $$\begin{array}{c}{\text{Y}}_{\text{i}} \sim \text{ NB }({\upmu }_{\text{i}},\text{ r})\\ \text{E}({\text{Y}}_{\text{i}}) = {\upmu }_{\text{i}}\\ {\upmu }_{\text{i}}={\text{E}}_{\text{i}}\times {\theta }_{\text{i}}\end{array}$$

where μ_i_ is the mean of GHS cases in area i, and r is the dispersion parameter. The mean μ_i_ is the product of the expected cases E_i_ and the municipality-specific relative risk (RR) θ_i_. E_i_ was calculated using indirect age standardization and was included as an offset term in the statistical model. The RR θ_i_ represents how much higher or lower the risk of GHS is for a specific municipality compared to the overall average risk.

To model the RR, a log-linear model links θ_i_ to explanatory variables, covariates, and spatial random effects. The ICAR model includes only a spatially structured effect (S_i_), which allows information sharing among neighboring areas. The logarithm of the RR was modeled as:

2 $$\text{log}({\uptheta }_{\text{i}}) = \alpha+ \sum {\beta }_{\text{k}} {\text{X}}_{\text{ik}} + \sum {\beta }_{\text{j}} {\text{Z}}_{\text{ij}} + {\text{S}}_{\text{i}}$$

Here, α is the overall intercept, representing the logarithm of the baseline RR across all regions. β_k_ and β_j_ are the coefficients for the explanatory variables X_ik_ and covariates Z_ij_, respectively. S_i_ is the structured spatial random effect that accounts for spatial autocorrelation.

In contrast, the BYM model includes both a spatially structured (S_i_) and an unstructured (U_i_) random effect. The unstructured component accounts for spatial randomness or region-specific variation not explained by the covariates or spatial structure:

3 $$\text{log}\left({\uptheta }_{\text{i}}\right)= \alpha+ \sum {\beta }_{\text{k}} {\text{X}}_{\text{ik}} + \sum { \beta }_{\text{j}} {\text{Z}}_{\text{ij}} + {\text{S}}_{\text{i}}+{\text{U}}_{\text{i}}$$

Both the ICAR and BYM models were fitted to the data. The models were then compared using the Deviance Information Criterion (DIC) to identify the model that provided the better fit to the data [46].

All Bayesian hierarchical models were fitted using the R-INLA package in R (version 4.2.3) [47]. This package implements the integrated nested Laplace approximation (INLA) approach. Although INLA may offer fewer options for specifying complex likelihoods than Markov chain Monte Carlo (MCMC) methods, INLA serves as a computationally efficient alternative to MCMC, producing robust regression estimates in analyses of spatially autocorrelated data and providing substantial reductions in computation time [47, 48].

For the fixed effects (intercept and coefficients), default non-informative priors were assigned. For the hyperparameters of the structured (S_i_) and unstructured (U_i_) random effects, a sensitivity analysis was performed to compare five non-informative prior specifications (see Supplementary Table S1) [49]. The effect of spatial weights matrices was also evaluated by comparing k-nearest neighbors (k = 3–7) and Queen contiguity definitions [50]. These comparisons were based on the DIC from the BYM model [46]. Ultimately, the combination of log-Gamma priors for the precision parameters (τ_S_ ∼ log-Gamma (0.1, 0.1); τ_U_ ∼ log-Gamma (0.001, 0.001)) and k-nearest neighbors (k = 6) spatial matrix was selected as it minimized the DIC, and this configuration was adopted for all subsequent analyses.

A two-stage variable selection process was employed to build the final multivariable model. First, bivariate screening was conducted. Each candidate explanatory variable was individually included in the bivariate BYM model, and its significance was assessed. A variable was retained for the next stage if the 80% posterior credible interval (CrI) for its RR (i.e., the exponentiated coefficient, exp(β)) did not contain the null value of 1.0. This less restrictive 80% threshold, as opposed to the conventional 95% interval, was intentionally chosen to minimize the risk of prematurely excluding potentially important variables during the screening phase [51]. In the second stage, multicollinearity among the selected variables was assessed using the variance inflation factor (VIF). No variable exceeded the VIF threshold of 5 [52], indicating that multicollinearity was not a significant issue in the final model.

To visualize the spatial distribution of GHS risk in each municipality, the posterior means were extracted from R-INLA output using the best-fit model [53]. Municipalities whose 95% CrI for RR excluded 1 were classified as having significantly elevated risk (RR > 1) or significantly reduced risk (RR < 1); all others were considered non‐significant. The risk map was visualized using the'tmap'package in R. [41].

## Results

### Descriptive analysis

In 2019, KNHIS data identified 182,289 cases of GHS in South Korea. The incidence rates peaked among adults aged 30–39 years (548 per 100,000), followed by those aged 20–29 years (544 per 100,000). The third-highest incidence was among adults aged 50–59 years (413 per 100,000). The incidence was lower among children aged under 9 years (< 10 per 100,000), adolescents aged 10–19 years (55 per 100,000), and adults aged over 80 years (< 150 per 100,000) (Figure S2). The GHSsir varied from 58 to 705 per 100,000 people across municipalities, a more than 12-fold difference (Table 2).

**Table 2:** Summary of standardized genital herpes simplex incidence rates and regional risk factor distributions across 250 South Korean municipalities in 2019

Variables	Mean	SD	Minimum	Median	Maximum
Cases of genital herpes simplex (N)	727.61	675.02	19	523	3,348
Standardized incidence rate of genital herpes simplex (per 100,000 people)	320.51	94.68	58.27	320.41	705.38
Health behavior					
Exercise and physical activities (%)	25.36	6.88	5.50	24.75	56.90
Current smoking (%)	20.27	3.09	11.60	20.25	28.80
Alcohol consumption (%)	59.28	4.36	44.80	59.90	71.00
High-risk drinking (%)	14.16	2.98	5.80	14.10	24.80
Welfare-related variables					
Social welfare facilities density (per 100,000 people)	19.12	11.83	2.3	16.65	73.70
Share of municipal budget on social welfare (%)	34.70	14.71	11.40	32.90	66.60
Subjective health perception (%)	42.64	6.98	29.70	41.40	68.30
EQ-5D index	0.96	0.01	0.93	0.96	0.99
Healthy living practice (%)	29.02	9.48	9.60	28.50	54.70
Health conditions					
Prevalence of depression (%)	3.04	1.38	0.40	2.90	7.20
Prevalence of obesity (%)	34.38	3.72	24.30	34.25	44.80
Prevalence of diabetes (%)	8.04	1.32	5.00	8.00	11.80
Prevalence of hypertension (%)	19.44	2.28	14.90	19.30	27.40
Socioeconomic variables					
Low educational attainment (%)	51.39	11.65	14.84	52.32	73.13
Single-person household (%)	23.90	5.50	12.99	23.80	39.05
Divorce (%)	2.18	0.42	1.20	2.20	4.10
Financial autonomy (%)	25.16	14.01	7.05	20.93	68.90
Healthcare accessibility					
Doctors per 1,000 people	2.78	2.20	1.00	2.30	19.60
Unmet medical facilities (%)	6.48	3.40	0.90	6.00	19.10
Adult entertainment and sexual violence					
Adult entertainment establishments per 10,000 people	7.70	6.53	0.11	6.32	64.29
Sexual violence risk	99.95	18.22	68.53	96.22	203.78
Covariates					
Population density (N/1,000km^2^)	3.83	5.85	0.02	0.68	26.32
Sex ratio (%)	101.53	6.72	87.60	100.40	134.70

**Fig. 1:**
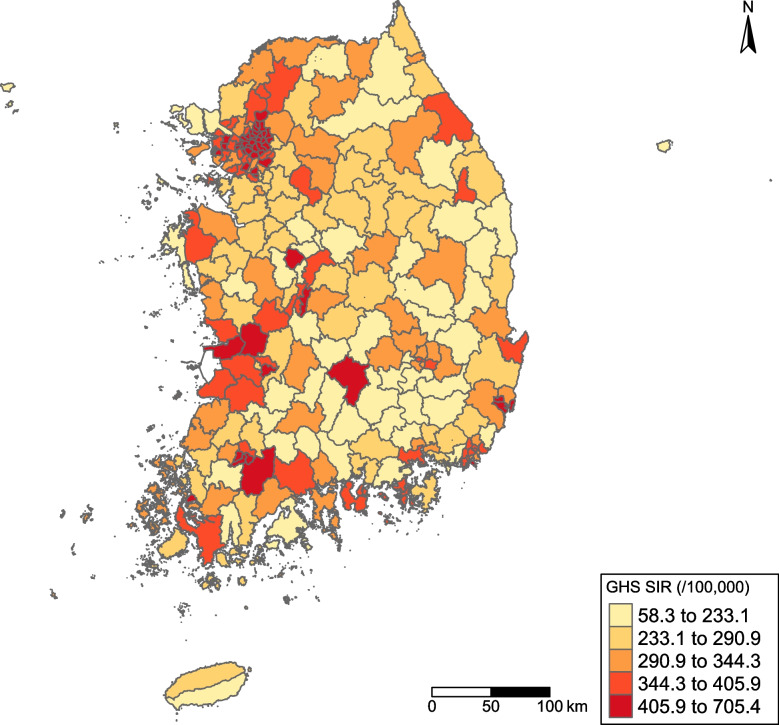
Geographic distribution of standardized genital herpes simplex incidence rates (per 100,000 population) across 250 South Korean municipalities in 2019. Municipalities are color-coded from yellow (low incidence) to red (high incidence)

The choropleth map (Fig. 1) revealed substantial geographic variation in GHSsir across the 250 municipalities. The highest rates were concentrated in the Seoul Capital Area (Seoul, Incheon, and Gyeonggi). The central, southern, and eastern regions had notably lower incidence rates, with many municipalities reporting < 233 cases per 100,000. Global Moran's I indicated spatial autocorrelation (I = 0.356, *p* < 0.001), suggesting that municipalities with similar incidence rates tend to cluster.

**Fig. 2:**
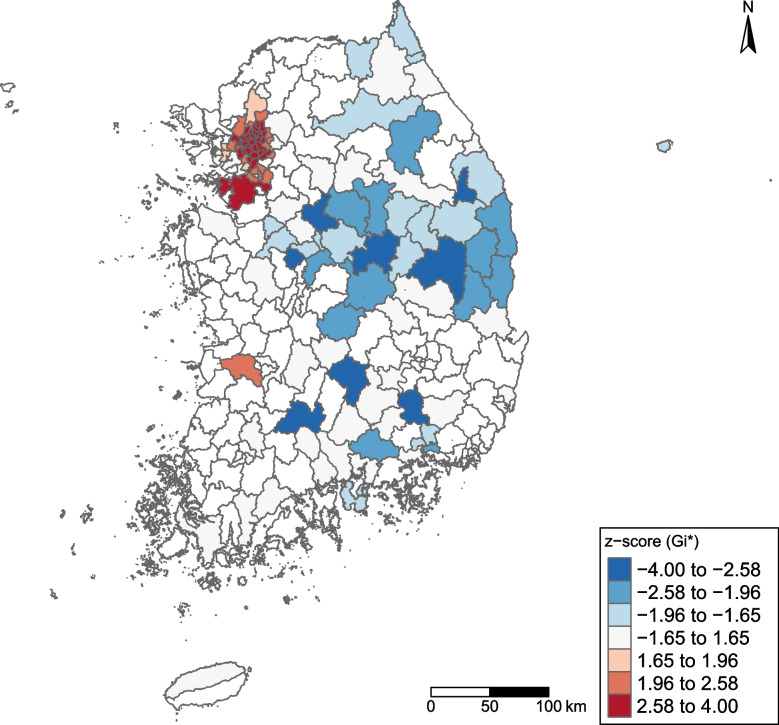
Hot spot analysis of standardized genital herpes simplex incidence rates across South Korea in 2019 using the Getis–Ord Gi*. Red areas (high positive z-scores) indicate significant hot spots, whereas blue areas (low negative z-scores) indicate significant cold spots

The Getis–Ord Gi* analysis revealed significant hot and cold spots (Fig. 2). The hot spots were concentrated in the Seoul Capital Area, with 43 municipalities showing significantly high incidence rates (*p* < 0.05). The cold spots (20 municipalities) were scattered in the central, southern, and eastern regions (*p* < 0.05).

### Bayesian hierarchical models

In the Bayesian hierarchical analysis, the BYM model provided the best fit, with a lower DIC (2691.735) than the ICAR model (DIC = 2939.050, Table 3). In the BYM analysis adjusting for all covariates and spatial effects, two area-level factors showed statistically significant associations with GHSsir (Table 3).

**Table 3:** Associations between municipal-level risk factors and standardized genital herpes simplex incidence rate, as estimated by Bayesian spatial models (ICAR vs BYM), South Korea, 2019

Variables^a^	ICAR^b^	BYM^c^
Alcohol consumption (%)	1.001 (0.993–1.009)	1.000 (0.992–1.009)
Healthy living practice (%)	1.004 (0.999–1.008)	1.004 (0.999–1.009)
Share of municipal budget on social welfare (%)	1.003 (0.999–1.006)	1.003 (0.999–1.007)
Social welfare facilities density (per 100,000 people)	1.000 (0.997–1.003)	1.000 (0.997–1.003)
Prevalence of obesity (%)	0.995 (0.987–1.004)	0.995 (0.987–1.005)
Low educational attainment (%)	1.002 (0.998–1.006)	1.002 (0.998–1.007)
Single-person household (%)	1.026 (1.017–1.034)	1.026 (1.017–1.035)
Divorce (%)	0.975 (0.889–1.071)	0.973 (0.883–1.072)
Doctors per 1,000 population	1.004 (0.989–1.019)	1.003 (0.988–1.018)
Adult entertainment establishments per 10,000 population	1.005 (0.999–1.010)	1.005 (0.999–1.010)
Sexual violence risk Q1	1.000 (ref)	1.000 (ref)
Sexual violence risk Q2	1.099 (1.015–1.190)	1.106 (1.018–1.201)
Sexual violence risk Q3	1.086 (0.999–1.181)	1.095 (1.003–1.195)
Sexual violence risk Q4	1.080 (0.977–1.193)	1.087 (0.980–1.206)
Sex ratio	0.996 (0.990–1.002)	0.996 (0.990–1.002)
Population density (per 1,000km^2^)	1.000 (0.992–1.009)	1.000 (0.991–1.009)
Dispersion parameter	33.98 (24.52–46.42)	129.79 (64.23–71,602.08)
Precision of spatially structured component	27.20 (9.36–65.62)	22.86 (8.43–52.63)
Precision of spatially unstructured component	–	34.39 (20.24–50.71)
DIC^d^	2,939.050	2,691.735

^a^Results are expressed as adjusted relative risk and 95% credible intervals

^b^*ICAR* Intrinsic Conditional Autoregressive model

^c^*BYM* Besag-York-Mollié model

^d^*DIC* Deviance information criterion

First, the proportion of single-person households was positively associated with higher GHS incidence rates (RR = 1.026, 95% CrI: 1.017–1.035), indicating that municipalities with more single-person households had a significantly greater risk of GHS. Second, the risk of sexual violence was associated with GHS incidence rates: municipalities in the second and third quartiles of sexual violence risk experienced higher incidence rates compared to those in the lowest quartile (Q2: RR = 1.106, 95% CrI: 1.018–1.201; Q3: RR = 1.095, 95% CrI: 1.003–1.195). The association for the highest quartile of sexual violence risk was not statistically significant.

**Fig. 3:**
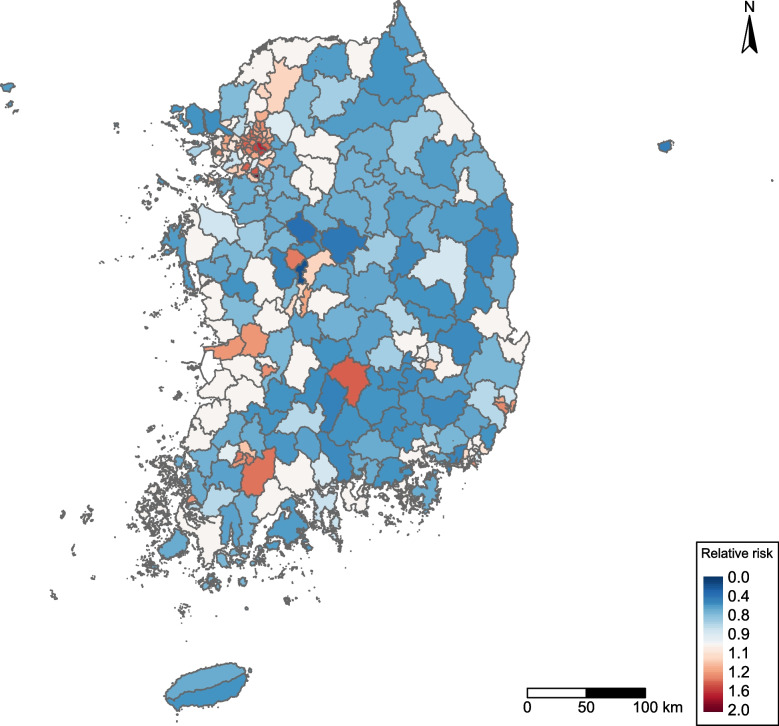
Municipality-level posterior mean relative risk (RR) of genital herpes simplex from the Besag–York–Mollié model in South Korea, 2019. Municipalities with significantly higher risk (RR > 1, 95% CrI excludes 1) are shown in red, and those with significantly lower risk (RR < 1) are shown in blue

Figure 3 illustrates the distribution of the municipality-specific relative risk of GHS (compared to the South Korea 2019 average), using the posterior mean from the BYM model. Significantly high-risk areas (shown in red in Fig. 3) were concentrated in the Seoul Capital Area and dispersed throughout several mid-southern cities. In contrast, significantly lower-risk areas (blue shading) were scattered throughout the country.

## Discussion

This study provided a nationwide spatial analysis of GHS incidence rates in South Korea. There was marked spatial heterogeneity in GHS incidence rates across the country, with distinct high-risk clusters identified by the Getis-Ord Gi* model. Moreover, even after adjusting for multiple risk factors and spatial dependence using the BYM model, the same high-risk regions were identified in the Seoul Capital Area and substantial residual geographic variation in GHS risk persisted, suggesting that other unmeasured contextual characteristics contribute to GHS risk. A higher proportion of single-person households and greater risk of sexual violence at the municipality-level were significantly associated with an increased risk of GHS after adjusting for covariates and spatial dependence using the Bayesian hierarchical model. To our knowledge, this is the first nationwide spatial epidemiological study of GHS in South Korea, demonstrating distinct geographic clustering and identifying key municipality-level determinants that may drive these patterns.

Moran’s I = 0.431 (*p* < 0.001) indicates that neighboring municipalities share similar GHS burdens, highlighting the place-effects on disease transmission [54]. The geographic clustering of GHS observed in this study aligns with multiple studies demonstrating that STIs often exhibit pronounced spatial clustering at subnational levels [13, 55, 56]. Specifically, the high-risk cluster of GHS in the Seoul Capital Area (Figs. 2 and 3) mirrors patterns seen for other STIs globally, where core areas of transmission are often found in densely populated regions [57]. In the United States, county-level analyses have revealed that hot spot counties for chlamydia, gonorrhea, and syphilis were predominantly clustered in specific geographic regions [13]. Similarly, Law et al. reported that each STI had a primary core area of high incidence rates, and that these core areas overlapped between infections [56]. Our detection of specific high-risk clusters is also consistent with recent spatial analyses from other settings. A Chinese study of GHS from 2010–2023 found significant spatiotemporal clustering, with the number of high-incidence hotspot counties concentrated in certain provinces [58]. Although South Korea and China differ in context, both analyses suggested that GHS risk is geographically clustered rather than random. On the other hand, the identified cold spots were not the result of targeted prevention interventions but rather reflected low levels of the identified regional risk factors in this study. While these areas exhibited protective characteristics, continued surveillance and monitoring are essential to maintain their low incidence rates and prevent potential emergence of risk factors that could alter their epidemiological profile. Overall, these findings suggest that GHS prevention should consider geographic variations rather than uniform nationwide strategies [9, 10].

A key finding of this study was the significant positive association between the proportion of single-person households and GHS incidence rates. This finding aligns with existing individual-level research demonstrating that individuals living alone have higher STI risk compared to those cohabiting with others [59, 60]. Curtis et al. found that young women living alone or with non-relatives were significantly more likely to report sexual risk behaviors than those living with parents, independent of age and relationship status [60]. Similarly, a study in Hong Kong reported elevated STI prevalence among individuals living alone, suggesting that household structure influences sexual health outcomes across different Asian contexts [59]. However, contextual effects linking municipal-level single-person household proportions to STI incidence rates remain relatively rare in the literature. Previous spatial analyses of STI epidemiology have primarily focused on the effects of socioeconomic variables [28, 61], demonstrating that socioeconomically disadvantaged areas facilitate the formation of sexual networks that promote STI transmission. Importantly, our study found that single-person household proportions remained a significant risk factor for GHS incidence rates even after adjusting for socioeconomic variables (which were not statistically significant in our model), suggesting that household structure operates through mechanisms beyond traditional socioeconomic pathways. Given the continuing increase in single-person households in South Korea [62], such changes in household structure suggest evolving social contextual effects that may alter sexual network patterns and STI transmission dynamics [63]. Therefore, further research is needed to fully understand the underlying mechanisms through which the proportion of single-person households acts as a risk factor for GHS.

The positive association between sexual violence risk and GHS incidence rates suggests that place-based vulnerability beyond individual risk factors influences population-level transmission. Sexual violence at the individual level is a well-established risk factor for STIs including GHS, due to forced unprotected exposure and trauma-related barriers to accessing care [34, 64]. Our ecological findings align with multiple studies showing that incorporating violent crime rates can improve the explanation of geographic STI patterns, presumably by capturing difficult-to-measure social determinants [65, 66]. Chesson et al. provided compelling evidence for this relationship, showing consistent state-level correlations between violent crime rates and reported STI rates across three decades in the United States, supporting the use of violent crime rates as a proxy for social determinants of STI incidence rates [67]. Our analysis suggests that areas with higher sexual violence risk may reflect underlying social norms and structural factors that create a more permissive environment for both violence and sexual risk-taking. Taken together, these results highlight the need for multifaceted interventions that address not only individual behaviors but also the social context that drives increased GHS risk.

This study has several limitations. First, as an ecological analysis of aggregated municipal data, our results cannot be directly applied to individual‐level causality and are subject to the ecological fallacy. To mitigate the bias introduced by the differing age structures across the municipalities, we calculated the indirect age‐standardized incidence rates of GHS and used age-standardized rates of the KCHS-derived explanatory variables. This approach has been shown to reduce ecological bias in aggregate‐level regression models [68]. Second, although global and U.S. surveillance data report higher GHS incidence in women [1, 2], our spatial analysis did not examine sex‐specific patterns because sex‐stratified case counts in each municipality were unavailable. We instead adjusted for underlying sex differences by including municipality‐level sex ratio as a covariate. Future research should incorporate sex‐stratified spatial modeling to elucidate male–female differences in GHS risk [53]. Third, our analysis was cross-sectional and limited to a single year (2019). Using only one year of data could lead to instability in rates for areas with small populations and does not capture temporal trends. Future research should incorporate longitudinal data across multiple years to conduct comprehensive spatiotemporal modeling of GHS risk factors, confirming whether the spatial clusters we identified persist or change over time. Fourth, while the BYM model effectively accounted for spatial dependence, it assumed constant effects of independent variables across all municipalities. Future research could explore models that allow for spatially varying coefficients, such as Bayesian geographically weighted regression to more comprehensively analyze how the associations between risk factors and GHS incidence rates may differ by region [69]. Finally, our reliance on KNHIS data introduced inherent limitations. The data reflected diagnosed cases seeking medical care, and thus likely underestimated true incidence by missing asymptomatic individuals or those who do not seek medical care. Additionally, if healthcare-seeking behavior varies geographically, this could introduce spatial bias in our risk estimates. To address this potential confounding, we included two healthcare accessibility variables in our models, physician density and unmet medical needs rates. However, residual bias may still exist if other unmeasured factors related to healthcare-seeking behavior differ across municipalities. Despite these limitations, our analysis provided valuable population-level insights. The use of KNHIS data offered a representative, population-based GHS incidence rates for 2019. The application of the Bayesian hierarchical model allowed us to account for spatial dependency, enabling us to map the GHS risk for municipalities and yielding reliable estimates of the associations between area-level risk factors and GHS incidence rates.

## Conclusion

This study demonstrates that GHS incidence rates in South Korea exhibit significant and distinct geographic patterns, with clear spatial clustering in the Seoul Capital Area. The identification of area-level risk factors, specifically a higher proportion of single-person households and greater risk of sexual violence, highlights the importance of social contexts for increasing GHS risk. These findings underscore the limitations of uniform strategies for preventing GHS and support the need for interventions that are tailored to local contexts and targeted to specific geographic areas. Further research is needed to investigate the underlying mechanisms through which the identified contextual risk factors influence GHS transmission. Such mechanistic studies would provide crucial insights for developing effective policy interventions tailored to specific local contexts.

## Supplementary Information

Supplementary Material 1.

## Data Availability

No datasets were generated or analysed during the current study.

## Abbreviations

BYM: Besag–York–Mollié model
CrI: Credible interval
DIC: Deviance information criterion
GHS: Genital herpes simplex
HSV: Herpes simplex virus
ICAR: Intrinsic conditional autoregressive model
INLA: Integrated nested Laplace approximation
KCHS: Korea Community Health Survey
KNHIS: Korea National Health Insurance Service
KOSIS: Korea Statistical Information Service
MCMC: Markov chain Monte Carlo
RR: Relative risk
STI: Sexually transmitted infection
VIF: Variance inflation factor
