# Supplementary material for: How place shapes genital herpes simplex distribution in South Korea: a Bayesian spatial analysis using National Health Insurance Service data
Source: BMC Public Health. 2025 Sep 2;25:3023. doi: 10.1186/s12889-025-24171-4 (PMC12403936; doi:10.1186/s12889-025-24171-4)
Supplement: Supplementary file 1 — Supplementary Material 1. [file 12889_2025_24171_MOESM1_ESM.docx]

**Supplementary Information**

**Preliminary analysis of comparing spatial weight matrices and priors**

A preliminary analysis was performed to select the spatial weights matrix [1], comparing k-nearest neighbors (k = 3–7) and Queen contiguity based on DIC from the Besag-York-Mollié (BYM) models [2]. Five non-informative priors for spatial (τ_S_) and non-spatial (τ_U_) random effects were evaluated [3].

Prior 1. τ_S_ ~log-Gamma (0.1,0.01), τ_U_ ~ log-Gamma (0.1,0.01)

Prior 2. τ_S_ ~log-Gamma (0.1,0.1), τ_U_ ~ log-Gamma (0.001,0.001)

Prior 3. τ_S_ ~log-Gamma (0.5,0.001), τ_U_ ~ log-Gamma (0.5,0.001)

Prior 4. τ_S_ ~log-Gamma (1,0.005), τ_U_ ~ log-Gamma (1,0.005)

Prior 5. τ_S_ ~ PC (0.5,0.01), τ_U_ ~ PC (0.5,0.01)

**Table S1.** Deviance Information Criterion of the BYM model with the combination of prior and weight matrix.

| Priors | KNN3 | KNN4 | KNN5 | KNN6 | KNN7 | Queen |
| --- | --- | --- | --- | --- | --- | --- |
| Prior1 | 3032.110 | 3005.812 | 2988.622 | 2985.409 | 2991.500 | 3014.791 |
| Prior2 | 2960.555 | 2752.383 | 2764.987 | 2747.458 | 2758.152 | 2881.827 |
| Prior3 | 3047.303 | 3033.546 | 3029.630 | 3031.904 | 3033.472 | 3039.645 |
| Prior4 | 3047.053 | 3032.526 | 3027.895 | 3030.614 | 3032.673 | 3037.662 |
| Prior5 | 3047.902 | 3034.615 | 3030.735 | 3034.165 | 3041.155 | 3039.840 |

Note: BYM, Besag-York-Mollié model; KNN, k-nearest neighbor


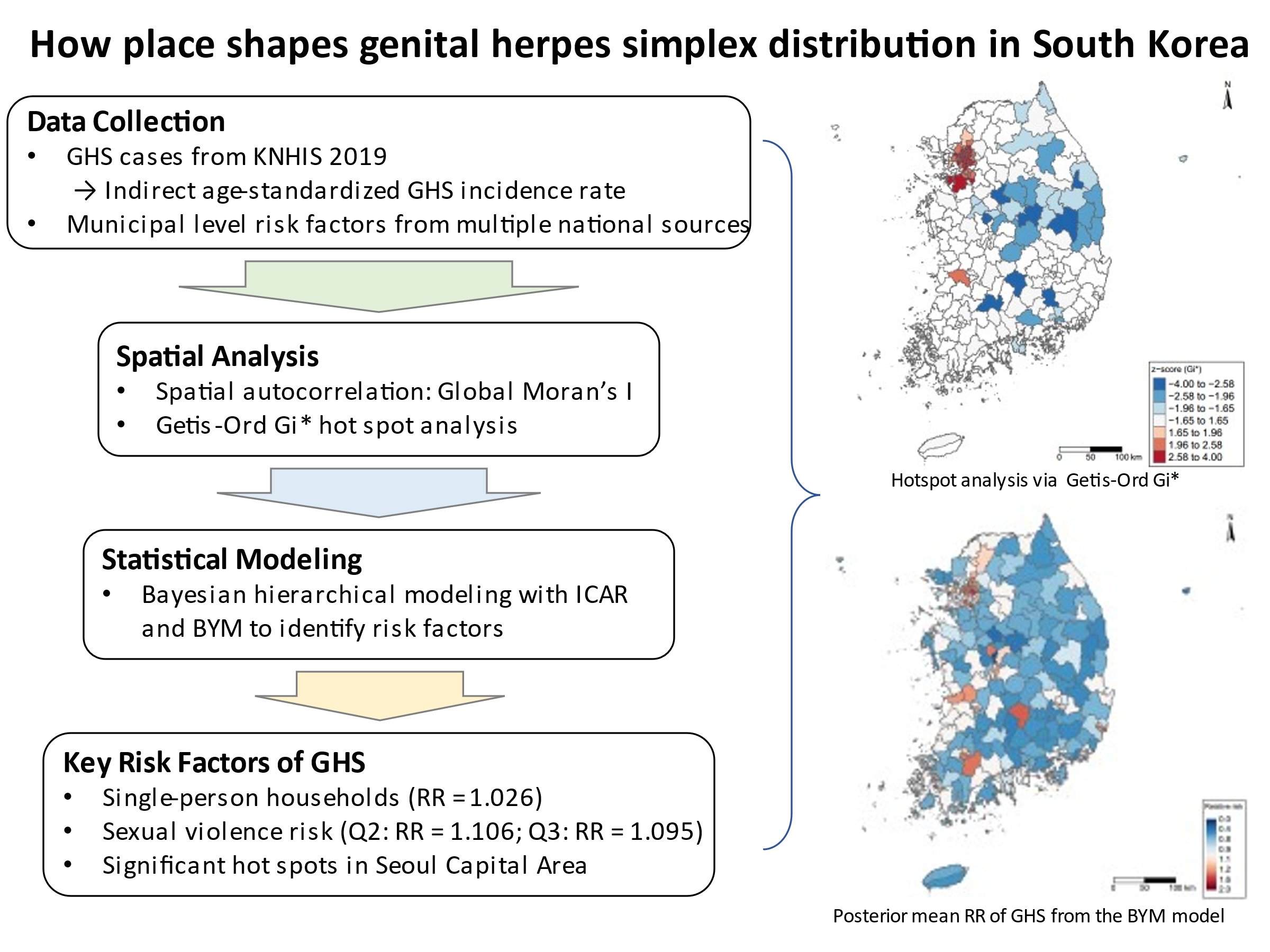


**Figure S1**. Overall workflow of the municipal-level spatial analysis of genital herpes simplex in South Korea, 2019. GHS, genital herpes simplex; KNHIS, Korean National Health Insurance Service; ICAR, the intrinsic conditional autoregressive model; BYM, the Besag–York–Mollié model; RR, relative risk

**Figure S2**. The genital herpes simplex incidence rates per 100,000 population by age group in South Korea, 2019.

**References**

1. Cortes-Ramirez J, Vilcins D, Jagals P, Soares Magalhaes RJ. Environmental and sociodemographic risk factors associated with environmentally transmitted zoonoses hospitalisations in Queensland, Australia. One Health. 2021;12:100206. <https://doi.org/10.1016/j.onehlt.2020.100206>.

2. Spiegelhalter DJ, Best NG, Carlin BP, Van Der Linde A. Bayesian measures of model complexity and fit. Journal of the Royal Statistical Society: Series B (Statistical Methodology). 2002;64:583-639. <https://doi.org/https://doi.org/10.1111/1467-9868.00353>.

3. Cortes-Ramirez J, Wilches-Vega JD, Caicedo-Velasquez B, Paris-Pineda OM, Sly PD. Spatiotemporal hierarchical Bayesian analysis to identify factors associated with COVID-19 in suburban areas in Colombia. Heliyon. 2024;10:e30182. <https://doi.org/10.1016/j.heliyon.2024.e30182>.
